# 3,19-Diacetyl-12-nitro­methyl-14-deoxy­andrographolide

**DOI:** 10.1107/S160053680904402X

**Published:** 2009-10-31

**Authors:** Juan Jia, Cheng Yao

**Affiliations:** a College of Science, Nanjing University of Technolgy, Xinmofan Road No. 5 Nanjing, Nanjing 210009, People’s Republic of China

## Abstract

In the crystal of the title compound, C_24_H_33_NO_9_, inter­molecular C—H⋯O hydrogen bonds link the mol­ecules.

## Related literature

For general background, see: Thunuguntla *et al.* (2004 ▶). For bond-length data, see: Allen *et al.* (1987 ▶). 
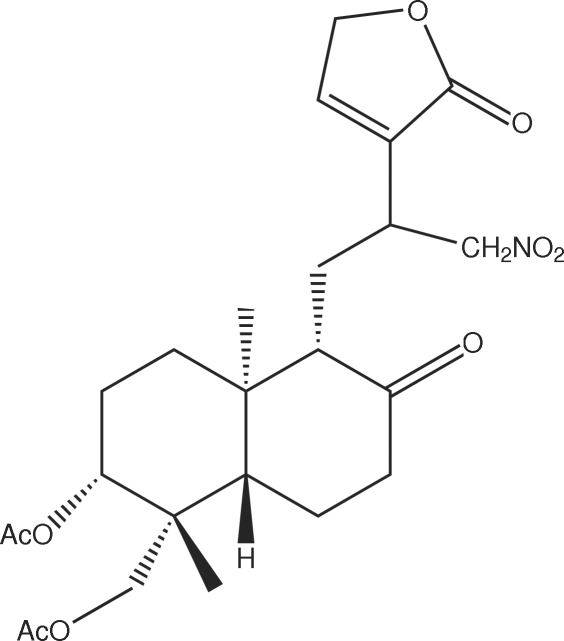


## Experimental

### 

#### Crystal data

C_24_H_33_NO_9_
*M* *_r_* = 479.51
Monoclinic, 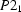

*a* = 10.533 (2) Å
*b* = 12.756 (3) Å
*c* = 10.659 (2) Å
β = 117.04 (3)°
*V* = 1275.6 (4) Å^3^
*Z* = 2
Mo *K*α radiation
μ = 0.10 mm^−1^
*T* = 293 K
0.30 × 0.20 × 0.20 mm

#### Data collection

Enraf–Nonius CAD-4 diffractometer
Absorption correction: none
2565 measured reflections
2431 independent reflections
1945 reflections with *I* > 2σ(*I*)
*R*_int_ = 0.024
3 standard reflections every 200 reflections intensity decay: 1%

#### Refinement

*R*[*F* ^2^ > 2σ(*F* ^2^)] = 0.072
*wR*(*F* ^2^) = 0.199
*S* = 1.00
2431 reflections
302 parameters
14 restraints
H-atom parameters constrained
Δρ_max_ = 0.69 e Å^−3^
Δρ_min_ = −0.48 e Å^−3^

### 

Data collection: *CAD-4 Software* (Enraf–Nonius, 1989 ▶); cell refinement: *CAD-4 Software*; data reduction: *XCAD4* (Harms & Wocadlo, 1995 ▶); program(s) used to solve structure: *SHELXS97* (Sheldrick, 2008 ▶); program(s) used to refine structure: *SHELXL97* (Sheldrick, 2008 ▶); molecular graphics: *SHELXTL* (Sheldrick, 2008 ▶); software used to prepare material for publication: *SHELXL97*.

## Supplementary Material

Crystal structure: contains datablocks global, I. DOI: 10.1107/S160053680904402X/hb5144sup1.cif

Structure factors: contains datablocks I. DOI: 10.1107/S160053680904402X/hb5144Isup2.hkl

Additional supplementary materials:  crystallographic information; 3D view; checkCIF report

## Figures and Tables

**Table 1:** Hydrogen-bond geometry (Å, °)

*D*—H⋯*A*	*D*—H	H⋯*A*	*D*⋯*A*	*D*—H⋯*A*
C15—H15*A*⋯O8^i^	0.97	2.59	3.312 (18)	131
C22—H22*A*⋯O9^ii^	0.97	2.47	3.388 (13)	158
C22—H22*B*⋯O1^iii^	0.97	2.51	3.301 (13)	139
C24—H24*B*⋯O3^iv^	0.97	2.56	3.376 (9)	142

Symmetry codes: (i) 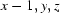; (ii) 
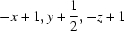
; (iii) 
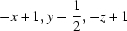
; (iv) 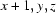.

**Fig. 1.**
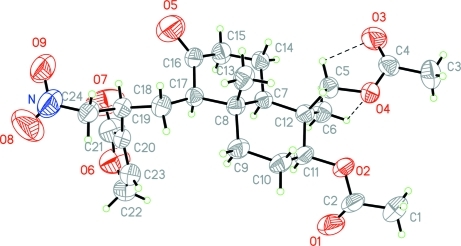
The molecular structure of (I) showing 30% displacement ellipsoids.

## supplementary crystallographic information

### Experimental

Andrographolide (15 g) and nitromethane (20 ml) in dry methanol were stired in the presence of sodium methoxide at room temperature for 3 h. After confirming the completion of reaction, the mixture was washed with brine. The organic phase was evaporated in vacuo, and the residue was recrystallized by ethyl acrtate. The product (10 g) and acetic anhydride (20 ml) was refluxed for 10 min. After confirming the completion of reaction, the mixture was washed with brine. The organic phase was evaporated in vacuo to afford corresponding product by flash chromatography.  Colourless blocks of (I) were recrystallised from ethyl acetate.

### Refinement

The H atoms were positioned geometrically  (C—H = 0.93–0.97Å) and refined as riding with U_iso_(H) = 1.2U_eq_(C) or 1.5U_eq_(methyl C).

The deepest difference hole is 0.12Å from the N atom and the highest difference peak is 0.18Å from atom O9.

### Crystal data

**Table:**

C_24_H_33_NO_9_	*F*(000) = 512
*M**_r_* = 479.51	*D*_x_ = 1.248 Mg m^−^^3^
Monoclinic, *P*2_1_	Mo *K*α radiation, λ = 0.71073 Å
Hall symbol: P 2yb	Cell parameters from 25 reflections
*a* = 10.533 (2) Å	θ = 9–12°
*b* = 12.756 (3) Å	µ = 0.10 mm^−^^1^
*c* = 10.659 (2) Å	*T* = 293 K
β = 117.04 (3)°	Block, colourless
*V* = 1275.6 (4) Å^3^	0.30 × 0.20 × 0.20 mm
*Z* = 2	

### Data collection

**Table:**

Enraf–Nonius CAD-4 diffractometer	*R*_int_ = 0.024
Radiation source: fine-focus sealed tube	θ_max_ = 25.3°, θ_min_ = 2.2°
graphite	*h* = −11→12
ω/2θ scans	*k* = −15→0
2565 measured reflections	*l* = −12→0
2431 independent reflections	3 standard reflections every 200 reflections
1945 reflections with *I* > 2σ(*I*)	intensity decay: 1%

### Refinement

**Table:**

Refinement on *F*^2^	Secondary atom site location: difference Fourier map
Least-squares matrix: full	Hydrogen site location: inferred from neighbouring sites
*R*[*F*^2^ > 2σ(*F*^2^)] = 0.072	H-atom parameters constrained
*wR*(*F*^2^) = 0.199	*w* = 1/[σ^2^(*F*_o_^2^) + (0.1*P*)^2^ + 1.120*P*] where *P* = (*F*_o_^2^ + 2*F*_c_^2^)/3
*S* = 1.00	(Δ/σ)_max_ < 0.001
2431 reflections	Δρ_max_ = 0.69 e Å^−^^3^
302 parameters	Δρ_min_ = −0.48 e Å^−^^3^
14 restraints	Extinction correction: *SHELXL97* (Sheldrick, 2008), Fc^*^=kFc[1+0.001xFc^2^λ^3^/sin(2θ)]^-1/4^
Primary atom site location: structure-invariant direct methods	Extinction coefficient: 0.063 (8)

### Special details

**Table:**

Geometry. All esds (except the esd in the dihedral angle between two l.s. planes) are estimated using the full covariance matrix. The cell esds are taken into account individually in the estimation of esds in distances, angles and torsion angles; correlations between esds in cell parameters are only used when they are defined by crystal symmetry. An approximate (isotropic) treatment of cell esds is used for estimating esds involving l.s. planes.
Refinement. Refinement of F^2^ against ALL reflections. The weighted R-factor wR and goodness of fit S are based on F^2^, conventional R-factors R are based on F, with F set to zero for negative F^2^. The threshold expression of F^2^ > 2sigma(F^2^) is used only for calculating R-factors(gt) etc. and is not relevant to the choice of reflections for refinement. R-factors based on F^2^ are statistically about twice as large as those based on F, and R- factors based on ALL data will be even larger.

### Fractional atomic coordinates and isotropic or equivalent isotropic displacement parameters (Å^2^)

**Table:**

	*x*	*y*	*z*	*U*_iso_*/*U*_eq_	
N	0.7992 (9)	−0.3512 (6)	0.7140 (12)	0.1177 (19)	
O1	0.1814 (7)	0.3398 (4)	0.6418 (6)	0.0915 (17)	
C1	0.0804 (12)	0.4065 (7)	0.7867 (12)	0.108 (3)	
H1A	0.0769	0.4728	0.7427	0.162*	
H1B	0.1387	0.4127	0.8866	0.162*	
H1C	−0.0143	0.3859	0.7679	0.162*	
O2	0.1466 (6)	0.2330 (3)	0.7887 (5)	0.0719 (13)	
C2	0.1425 (7)	0.3259 (5)	0.7293 (8)	0.0645 (16)	
O3	−0.1429 (6)	−0.0145 (5)	0.8828 (7)	0.0935 (18)	
C3	−0.1806 (8)	0.1612 (7)	0.9391 (9)	0.084 (2)	
H3A	−0.2668	0.1342	0.9365	0.126*	
H3B	−0.2033	0.2160	0.8704	0.126*	
H3C	−0.1207	0.1887	1.0312	0.126*	
O4	0.0165 (4)	0.1100 (3)	0.9106 (4)	0.0555 (10)	
C4	−0.1040 (7)	0.0749 (5)	0.9066 (6)	0.0587 (15)	
C5	0.1112 (7)	0.0355 (5)	0.8929 (6)	0.0559 (14)	
H5A	0.0813	−0.0350	0.9011	0.067*	
H5B	0.2070	0.0454	0.9681	0.067*	
C6	0.1141 (6)	0.0463 (5)	0.7499 (5)	0.0470 (12)	
O5	0.3609 (8)	−0.3524 (5)	0.7817 (8)	0.115 (2)	
C7	0.1806 (6)	−0.0527 (4)	0.7167 (5)	0.0424 (12)	
H7A	0.1659	−0.0412	0.6201	0.051*	
O6	0.4623 (8)	−0.1819 (6)	0.3207 (5)	0.106 (2)	
C8	0.3456 (6)	−0.0689 (4)	0.8036 (5)	0.0456 (12)	
O7	0.4337 (11)	−0.3352 (6)	0.4088 (7)	0.145 (3)	
O8	0.8651 (14)	−0.3399 (8)	0.6600 (15)	0.218 (7)	
C9	0.4175 (7)	0.0340 (5)	0.7949 (8)	0.0644 (16)	
H9A	0.5187	0.0294	0.8580	0.077*	
H9B	0.4063	0.0420	0.6999	0.077*	
O9	0.7740 (7)	−0.4391 (5)	0.7441 (9)	0.1177 (19)	
C10	0.3570 (7)	0.1318 (5)	0.8330 (8)	0.0655 (17)	
H10A	0.3776	0.1280	0.9313	0.079*	
H10B	0.4038	0.1937	0.8209	0.079*	
C11	0.2003 (7)	0.1420 (4)	0.7444 (7)	0.0558 (14)	
H11A	0.1817	0.1527	0.6465	0.067*	
C12	−0.0425 (6)	0.0581 (5)	0.6332 (6)	0.0596 (15)	
H12A	−0.0433	0.0659	0.5433	0.089*	
H12B	−0.0848	0.1188	0.6523	0.089*	
H12C	−0.0959	−0.0032	0.6321	0.089*	
C13	0.3944 (7)	−0.0991 (5)	0.9598 (6)	0.0595 (15)	
H13A	0.3676	−0.0447	1.0053	0.089*	
H13B	0.4961	−0.1075	1.0066	0.089*	
H13C	0.3498	−0.1637	0.9640	0.089*	
C14	0.0996 (6)	−0.1543 (4)	0.7078 (6)	0.0522 (13)	
H14A	−0.0017	−0.1426	0.6505	0.063*	
H14B	0.1150	−0.1743	0.8014	0.063*	
C15	0.1489 (7)	−0.2431 (5)	0.6436 (7)	0.0616 (16)	
H15A	0.1009	−0.3075	0.6453	0.074*	
H15B	0.1234	−0.2267	0.5462	0.074*	
C16	0.3051 (6)	−0.2581 (5)	0.7227 (6)	0.0524 (13)	
C17	0.3904 (6)	−0.1603 (4)	0.7310 (6)	0.0467 (12)	
H17A	0.3592	−0.1379	0.6334	0.056*	
C18	0.5504 (6)	−0.1759 (5)	0.7935 (6)	0.0554 (13)	
H18A	0.5837	−0.2095	0.8847	0.066*	
H18B	0.5959	−0.1078	0.8086	0.066*	
C19	0.5979 (6)	−0.2425 (5)	0.7016 (6)	0.0526 (14)	
H19A	0.5479	−0.3098	0.6836	0.063*	
C20	0.5592 (6)	−0.1911 (5)	0.5610 (6)	0.0524 (13)	
C21	0.4825 (11)	−0.2478 (8)	0.4296 (8)	0.087 (2)	
C22	0.5278 (9)	−0.0844 (7)	0.3765 (9)	0.088 (2)	
H22A	0.4587	−0.0279	0.3415	0.105*	
H22B	0.6036	−0.0701	0.3511	0.105*	
C23	0.5853 (8)	−0.0952 (6)	0.5302 (8)	0.0693 (17)	
H23A	0.6326	−0.0428	0.5957	0.083*	
C24	0.7548 (7)	−0.2639 (5)	0.7812 (8)	0.0649 (16)	
H24A	0.7784	−0.2830	0.8774	0.078*	
H24B	0.8072	−0.2008	0.7838	0.078*	

### Atomic displacement parameters (Å^2^)

**Table:**

	*U*^11^	*U*^22^	*U*^33^	*U*^12^	*U*^13^	*U*^23^
N	0.109 (3)	0.059 (2)	0.196 (6)	0.008 (3)	0.079 (4)	−0.003 (3)
O1	0.128 (5)	0.059 (3)	0.100 (4)	0.001 (3)	0.062 (4)	0.019 (3)
C1	0.147 (8)	0.051 (4)	0.152 (9)	0.012 (5)	0.090 (7)	0.001 (5)
O2	0.112 (4)	0.036 (2)	0.090 (3)	0.001 (2)	0.066 (3)	0.002 (2)
C2	0.068 (4)	0.037 (3)	0.083 (4)	0.001 (3)	0.030 (3)	0.007 (3)
O3	0.114 (4)	0.073 (3)	0.120 (4)	−0.029 (3)	0.077 (4)	−0.020 (3)
C3	0.072 (4)	0.097 (6)	0.093 (5)	0.009 (4)	0.046 (4)	−0.015 (5)
O4	0.066 (2)	0.047 (2)	0.062 (2)	−0.0022 (19)	0.037 (2)	−0.0078 (18)
C4	0.069 (4)	0.061 (4)	0.054 (3)	−0.009 (3)	0.035 (3)	−0.007 (3)
C5	0.072 (4)	0.055 (3)	0.051 (3)	0.006 (3)	0.036 (3)	−0.001 (3)
C6	0.061 (3)	0.043 (3)	0.040 (3)	−0.001 (3)	0.026 (2)	0.000 (2)
O5	0.157 (6)	0.081 (4)	0.128 (5)	0.010 (4)	0.082 (5)	0.007 (4)
C7	0.049 (3)	0.044 (3)	0.036 (2)	−0.002 (2)	0.021 (2)	−0.001 (2)
O6	0.155 (6)	0.095 (4)	0.065 (3)	−0.007 (4)	0.048 (3)	0.000 (3)
C8	0.051 (3)	0.043 (3)	0.044 (3)	−0.002 (2)	0.022 (2)	−0.005 (2)
O7	0.241 (9)	0.085 (5)	0.084 (4)	−0.049 (6)	0.054 (5)	−0.028 (4)
O8	0.294 (13)	0.122 (7)	0.410 (18)	−0.038 (8)	0.310 (15)	−0.057 (9)
C9	0.062 (3)	0.044 (3)	0.092 (5)	−0.008 (3)	0.039 (3)	−0.005 (3)
O9	0.109 (3)	0.059 (2)	0.196 (6)	0.008 (3)	0.079 (4)	−0.003 (3)
C10	0.075 (4)	0.038 (3)	0.088 (5)	−0.010 (3)	0.040 (4)	−0.011 (3)
C11	0.078 (4)	0.036 (3)	0.062 (3)	0.002 (3)	0.040 (3)	0.000 (3)
C12	0.065 (3)	0.058 (4)	0.053 (3)	0.008 (3)	0.023 (3)	0.002 (3)
C13	0.072 (4)	0.060 (4)	0.044 (3)	0.009 (3)	0.023 (3)	−0.006 (3)
C14	0.053 (3)	0.048 (3)	0.061 (3)	−0.003 (3)	0.030 (3)	−0.007 (3)
C15	0.059 (4)	0.054 (4)	0.073 (4)	−0.010 (3)	0.031 (3)	−0.019 (3)
C16	0.065 (4)	0.044 (3)	0.056 (3)	0.001 (3)	0.034 (3)	−0.007 (2)
C17	0.051 (3)	0.045 (3)	0.046 (3)	0.003 (2)	0.024 (2)	−0.001 (2)
C18	0.056 (3)	0.054 (3)	0.054 (3)	0.003 (3)	0.023 (3)	−0.003 (3)
C19	0.053 (3)	0.045 (3)	0.062 (3)	0.004 (3)	0.029 (3)	0.001 (3)
C20	0.055 (3)	0.044 (3)	0.066 (3)	0.007 (3)	0.034 (3)	0.001 (3)
C21	0.114 (6)	0.082 (6)	0.069 (5)	−0.005 (5)	0.044 (4)	−0.006 (4)
C22	0.104 (6)	0.083 (6)	0.099 (5)	0.020 (5)	0.066 (5)	0.033 (5)
C23	0.076 (4)	0.059 (4)	0.085 (5)	0.003 (3)	0.048 (4)	0.000 (3)
C24	0.058 (4)	0.047 (3)	0.085 (4)	0.006 (3)	0.029 (3)	0.005 (3)

### Geometric parameters (Å, °)

**Table:**

N—O8	1.094 (10)	C9—H9B	0.9700
N—O9	1.228 (10)	C10—C11	1.488 (9)
N—C24	1.508 (10)	C10—H10A	0.9700
O1—C2	1.190 (8)	C10—H10B	0.9700
C1—C2	1.492 (11)	C11—H11A	0.9800
C1—H1A	0.9600	C12—H12A	0.9600
C1—H1B	0.9600	C12—H12B	0.9600
C1—H1C	0.9600	C12—H12C	0.9600
O2—C2	1.335 (7)	C13—H13A	0.9600
O2—C11	1.460 (7)	C13—H13B	0.9600
O3—C4	1.200 (8)	C13—H13C	0.9600
C3—C4	1.495 (10)	C14—C15	1.531 (8)
C3—H3A	0.9600	C14—H14A	0.9700
C3—H3B	0.9600	C14—H14B	0.9700
C3—H3C	0.9600	C15—C16	1.481 (9)
O4—C4	1.327 (7)	C15—H15A	0.9700
O4—C5	1.451 (7)	C15—H15B	0.9700
C5—C6	1.544 (7)	C16—C17	1.517 (8)
C5—H5A	0.9700	C17—C18	1.518 (7)
C5—H5B	0.9700	C17—H17A	0.9800
C6—C11	1.538 (8)	C18—C19	1.541 (8)
C6—C12	1.557 (8)	C18—H18A	0.9700
C6—C7	1.561 (8)	C18—H18B	0.9700
O5—C16	1.361 (9)	C19—C24	1.500 (8)
C7—C14	1.530 (8)	C19—C20	1.512 (8)
C7—C8	1.568 (7)	C19—H19A	0.9800
C7—H7A	0.9800	C20—C23	1.328 (9)
O6—C21	1.369 (10)	C20—C21	1.452 (10)
O6—C22	1.416 (12)	C22—C23	1.473 (11)
C8—C9	1.539 (8)	C22—H22A	0.9700
C8—C13	1.551 (8)	C22—H22B	0.9700
C8—C17	1.585 (7)	C23—H23A	0.9300
O7—C21	1.205 (12)	C24—H24A	0.9700
C9—C10	1.538 (9)	C24—H24B	0.9700
C9—H9A	0.9700		
			
O8—N—O9	121.4 (10)	C6—C12—H12A	109.5
O8—N—C24	124.1 (9)	C6—C12—H12B	109.5
O9—N—C24	113.5 (9)	H12A—C12—H12B	109.5
C2—C1—H1A	109.5	C6—C12—H12C	109.5
C2—C1—H1B	109.5	H12A—C12—H12C	109.5
H1A—C1—H1B	109.5	H12B—C12—H12C	109.5
C2—C1—H1C	109.5	C8—C13—H13A	109.5
H1A—C1—H1C	109.5	C8—C13—H13B	109.5
H1B—C1—H1C	109.5	H13A—C13—H13B	109.5
C2—O2—C11	119.1 (5)	C8—C13—H13C	109.5
O1—C2—O2	123.8 (7)	H13A—C13—H13C	109.5
O1—C2—C1	126.2 (7)	H13B—C13—H13C	109.5
O2—C2—C1	110.1 (6)	C7—C14—C15	111.2 (5)
C4—C3—H3A	109.5	C7—C14—H14A	109.4
C4—C3—H3B	109.5	C15—C14—H14A	109.4
H3A—C3—H3B	109.5	C7—C14—H14B	109.4
C4—C3—H3C	109.5	C15—C14—H14B	109.4
H3A—C3—H3C	109.5	H14A—C14—H14B	108.0
H3B—C3—H3C	109.5	C16—C15—C14	110.9 (5)
C4—O4—C5	118.6 (5)	C16—C15—H15A	109.5
O3—C4—O4	123.9 (6)	C14—C15—H15A	109.5
O3—C4—C3	125.5 (7)	C16—C15—H15B	109.5
O4—C4—C3	110.7 (6)	C14—C15—H15B	109.5
O4—C5—C6	113.2 (5)	H15A—C15—H15B	108.1
O4—C5—H5A	108.9	O5—C16—C15	120.8 (6)
C6—C5—H5A	108.9	O5—C16—C17	125.5 (6)
O4—C5—H5B	108.9	C15—C16—C17	113.7 (5)
C6—C5—H5B	108.9	C16—C17—C18	115.3 (5)
H5A—C5—H5B	107.8	C16—C17—C8	109.5 (4)
C11—C6—C5	112.6 (5)	C18—C17—C8	113.6 (4)
C11—C6—C12	108.8 (5)	C16—C17—H17A	105.9
C5—C6—C12	108.0 (4)	C18—C17—H17A	105.9
C11—C6—C7	107.5 (4)	C8—C17—H17A	105.9
C5—C6—C7	111.3 (4)	C17—C18—C19	114.2 (5)
C12—C6—C7	108.6 (4)	C17—C18—H18A	108.7
C14—C7—C6	113.9 (4)	C19—C18—H18A	108.7
C14—C7—C8	111.2 (4)	C17—C18—H18B	108.7
C6—C7—C8	117.7 (4)	C19—C18—H18B	108.7
C14—C7—H7A	104.1	H18A—C18—H18B	107.6
C6—C7—H7A	104.1	C24—C19—C20	111.7 (5)
C8—C7—H7A	104.1	C24—C19—C18	109.5 (5)
C21—O6—C22	109.0 (6)	C20—C19—C18	111.9 (5)
C9—C8—C13	110.1 (5)	C24—C19—H19A	107.9
C9—C8—C7	107.2 (4)	C20—C19—H19A	107.9
C13—C8—C7	113.8 (4)	C18—C19—H19A	107.9
C9—C8—C17	109.5 (4)	C23—C20—C21	107.9 (6)
C13—C8—C17	108.3 (4)	C23—C20—C19	130.6 (6)
C7—C8—C17	107.7 (4)	C21—C20—C19	121.5 (6)
C10—C9—C8	113.7 (5)	O7—C21—O6	121.6 (8)
C10—C9—H9A	108.8	O7—C21—C20	129.9 (8)
C8—C9—H9A	108.8	O6—C21—C20	108.4 (7)
C10—C9—H9B	108.8	O6—C22—C23	105.1 (6)
C8—C9—H9B	108.8	O6—C22—H22A	110.7
H9A—C9—H9B	107.7	C23—C22—H22A	110.7
C11—C10—C9	112.1 (5)	O6—C22—H22B	110.7
C11—C10—H10A	109.2	C23—C22—H22B	110.7
C9—C10—H10A	109.2	H22A—C22—H22B	108.8
C11—C10—H10B	109.2	C20—C23—C22	109.5 (7)
C9—C10—H10B	109.2	C20—C23—H23A	125.2
H10A—C10—H10B	107.9	C22—C23—H23A	125.2
O2—C11—C10	110.2 (5)	C19—C24—N	111.5 (6)
O2—C11—C6	107.2 (4)	C19—C24—H24A	109.3
C10—C11—C6	114.2 (5)	N—C24—H24A	109.3
O2—C11—H11A	108.4	C19—C24—H24B	109.3
C10—C11—H11A	108.4	N—C24—H24B	109.3
C6—C11—H11A	108.4	H24A—C24—H24B	108.0
			
C11—O2—C2—O1	−0.7 (10)	C7—C14—C15—C16	55.1 (7)
C11—O2—C2—C1	178.3 (7)	C14—C15—C16—O5	122.3 (7)
C5—O4—C4—O3	−3.5 (9)	C14—C15—C16—C17	−57.0 (7)
C5—O4—C4—C3	175.1 (5)	O5—C16—C17—C18	8.8 (8)
C4—O4—C5—C6	108.6 (6)	C15—C16—C17—C18	−172.0 (5)
O4—C5—C6—C11	75.4 (6)	O5—C16—C17—C8	−120.8 (6)
O4—C5—C6—C12	−44.7 (7)	C15—C16—C17—C8	58.5 (6)
O4—C5—C6—C7	−163.8 (4)	C9—C8—C17—C16	−172.8 (5)
C11—C6—C7—C14	−175.9 (5)	C13—C8—C17—C16	67.1 (6)
C5—C6—C7—C14	60.3 (6)	C7—C8—C17—C16	−56.5 (5)
C12—C6—C7—C14	−58.4 (6)	C9—C8—C17—C18	56.7 (6)
C11—C6—C7—C8	51.4 (5)	C13—C8—C17—C18	−63.4 (6)
C5—C6—C7—C8	−72.4 (6)	C7—C8—C17—C18	173.0 (4)
C12—C6—C7—C8	168.9 (4)	C16—C17—C18—C19	67.9 (6)
C14—C7—C8—C9	174.9 (5)	C8—C17—C18—C19	−164.6 (5)
C6—C7—C8—C9	−51.2 (6)	C17—C18—C19—C24	−173.0 (5)
C14—C7—C8—C13	−63.1 (6)	C17—C18—C19—C20	62.6 (7)
C6—C7—C8—C13	70.8 (6)	C24—C19—C20—C23	−70.8 (8)
C14—C7—C8—C17	57.1 (5)	C18—C19—C20—C23	52.4 (8)
C6—C7—C8—C17	−169.0 (4)	C24—C19—C20—C21	108.2 (7)
C13—C8—C9—C10	−73.4 (7)	C18—C19—C20—C21	−128.6 (7)
C7—C8—C9—C10	50.9 (7)	C22—O6—C21—O7	178.3 (11)
C17—C8—C9—C10	167.6 (5)	C22—O6—C21—C20	2.0 (9)
C8—C9—C10—C11	−56.1 (8)	C23—C20—C21—O7	−176.9 (12)
C2—O2—C11—C10	89.9 (7)	C19—C20—C21—O7	3.9 (16)
C2—O2—C11—C6	−145.2 (5)	C23—C20—C21—O6	−0.9 (9)
C9—C10—C11—O2	177.3 (5)	C19—C20—C21—O6	179.9 (6)
C9—C10—C11—C6	56.5 (7)	C21—O6—C22—C23	−2.2 (9)
C5—C6—C11—O2	−51.7 (6)	C21—C20—C23—C22	−0.5 (8)
C12—C6—C11—O2	68.0 (6)	C19—C20—C23—C22	178.6 (6)
C7—C6—C11—O2	−174.6 (5)	O6—C22—C23—C20	1.7 (8)
C5—C6—C11—C10	70.7 (6)	C20—C19—C24—N	−70.7 (7)
C12—C6—C11—C10	−169.6 (5)	C18—C19—C24—N	164.7 (6)
C7—C6—C11—C10	−52.3 (6)	O8—N—C24—C19	110.7 (14)
C6—C7—C14—C15	167.4 (5)	O9—N—C24—C19	−80.2 (10)
C8—C7—C14—C15	−56.9 (6)		

### Hydrogen-bond geometry (Å, °)

**Table:**

*D*—H···*A*	*D*—H	H···*A*	*D*···*A*	*D*—H···*A*
C15—H15A···O8^i^	0.97	2.59	3.312 (18)	131
C22—H22A···O9^ii^	0.97	2.47	3.388 (13)	158
C22—H22B···O1^iii^	0.97	2.51	3.301 (13)	139
C24—H24B···O3^iv^	0.97	2.56	3.376 (9)	142

Symmetry codes: (i) *x*−1, *y*, *z*; (ii) −*x*+1, *y*+1/2, −*z*+1; (iii) −*x*+1, *y*−1/2, −*z*+1; (iv) *x*+1, *y*, *z*.

## References

[bb1] Allen, F. H., Kennard, O., Watson, D. G., Brammer, L., Orpen, A. G. & Taylor, R. (1987). *J. Chem. Soc. Perkin Trans. 2*, pp. S1–19.

[bb2] Enraf–Nonius (1989). *CAD-4 Software* Enraf–Nonius, Delft. The Netherlands.

[bb4] Harms, K. & Wocadlo, S. (1995). *XCAD4* University of Marburg, Germany.

[bb5] Sheldrick, G. M. (2008). *Acta Cryst.* A**64**, 112–122. 10.1107/S0108767307043930 18156677

[bb6] Thunuguntla, S. S. R., Nyavanandi, V. K. & Nanduri, S. (2004). *Tetrahedron Lett.* **45**, 9357–9360.

